# Human plasma metabolomics for identifying differential metabolites and predicting molecular subtypes of breast cancer

**DOI:** 10.18632/oncotarget.7155

**Published:** 2016-02-03

**Authors:** Yong Fan, Xin Zhou, Tian-Song Xia, Zhuo Chen, Jin Li, Qun Liu, Raphael N Alolga, Yan Chen, Mao-De Lai, Ping Li, Wei Zhu, Lian-Wen Qi

**Affiliations:** ^1^ State Key Laboratory of Natural Medicines, China Pharmaceutical University, Nanjing 210009, China; ^2^ Department of Oncology, The First Affiliated Hospital of Nanjing Medical University, Nanjing 210029, China; ^3^ Department of Breast Surgery, The First Affiliated Hospital of Nanjing Medical University, Nanjing 210029, China; ^4^ Emergency Center, The First Affiliated Hospital of Nanjing Medical University, Nanjing 210029, China

**Keywords:** human plasma metabolomics, differential metabolites, molecular subtypes, breast cancer

## Abstract

**Purpose:**

This work aims to identify differential metabolites and predicting molecular subtypes of breast cancer (BC).

**Methods:**

Plasma samples were collected from 96 BC patients and 79 normal participants. Metabolic profiles were determined by liquid chromatography-mass spectrometry and gas chromatography-mass spectrometry based on multivariate statistical data analysis.

**Results:**

We observed 64 differential metabolites between BC and normal group. Compared to human epidermal growth factor receptor 2 (HER2)-negative patients, HER2-positive group showed elevated aerobic glycolysis, gluconeogenesis, and increased fatty acid biosynthesis with reduced Krebs cycle. Compared with estrogen receptor (ER)-negative group, ER-positive patients showed elevated alanine, aspartate and glutamate metabolism, decreased glycerolipid catabolism, and enhanced purine metabolism. A panel of 8 differential metabolites, including carnitine, lysophosphatidylcholine (20:4), proline, alanine, lysophosphatidylcholine (16:1), glycochenodeoxycholic acid, valine, and 2-octenedioic acid, was identified for the classification of BC subtypes. These markers showed potential diagnostic value with average area under the curve at 0.925 (95% CI 0.867-0.983) for the training set (*n*=51) and 0.893 (95% CI 0.847-0.939) for the test set (*n*=45).

**Conclusion:**

Human plasma metabolomics is useful in identifying differential metabolites and predicting breast cancer subtypes.

## INTRODUCTION

Breast cancer (BC) is the most common cause of death among women worldwide [1]. Human epidermal growth factor receptor 2 (HER2), estrogen receptor (ER) are the two key molecular biomarkers to segregate the most distinct biologic subgroups of BC [2]. The characteristics of HER2 and ER can be used to roughly divide BC into four major molecular subtypes, including Luminal A (HER2 negative and ER positive), Luminal B (HER2 positive and ER positive), HER2-enriched (HER2 positive and ER negative), and Basal-Like (HER2 negative and ER positive) [3]. Each subtype of BC is accompanied with characteristic molecular features, subsequent metastatic lesions, prognosis and clinical responses to available medical therapies [4].

Determining the molecular subtype of BC is fundamental for personalized treatment. It was demonstrated that the “specific molecular type matched” patients had a higher overall response rate, longer time to treatment failure and longer survival compared to patients whose treatment was not matched to particular molecular abnormality [5]. Repeated biopsies and subsequent histopathology are commonly used to study molecular and genetic information from tumor cells for BC diagnosis and subtype classification. This analysis is invasive and time-consuming [6, 7]. Rapid and sensitive analysis is urgently required in clinic for discrimination of BC subtypes.

Recent studies have shown that genomic alterations in solid cancers can be characterized by bio-fluid metabolome change [8, 9]. Metabolomics is a new, rapidly expanding field dedicated to the global metabolic alterations in biological systems that occur in response to genetic, pathological, and environmental or lifestyle factors. The high-throughput nature of metabolomics makes it applicable to perform diagnostic biomarker screening for diseases or follow drug efficacy [10]. Plasma, a frequently considered pool of metabolites, has been applied to represent systemic metabolic deregulation in cancer patients, and the markers in this biological specimen could present biological mechanisms during cancer progression [9]. Metabolomics has been applied to find urinary biomarkers for BC [11]. Limited data, however, is available to characterize BC molecular subtypes by plasma metabolic profiles.

Gas chromatography coupled with mass spectrometry (GC-MS), liquid chromatography (LC)-MS, and nuclear magnetic resonance (NMR) are the three most commonly used platforms for metabolomic study [12, 13]. LC-MS is the most compatible technique for sensitive detection of biomolecules [14]. GC-MS technique provides a relatively more robust chromatography and greater separation efficiency together with the availability of reference compound libraries [13]. The parallel use of GC-MS and LC-MS could be a good choice to better profile different classes of compounds.

In this study, metabolomics was applied to identify differential metabolites and predicting molecular subtypes of breast cancer. We collected plasma samples from 96 BC patients and 79 normal control (NC) participants. Analysis was performed on ultra-performance liquid chromatography-quadrupole time of flight mass spectrometry (UPLC-Q/TOF-MS) and gas chromatography-quadrupole mass spectrometry (GC-Q/MS).

## RESULTS

**Figure 1:**
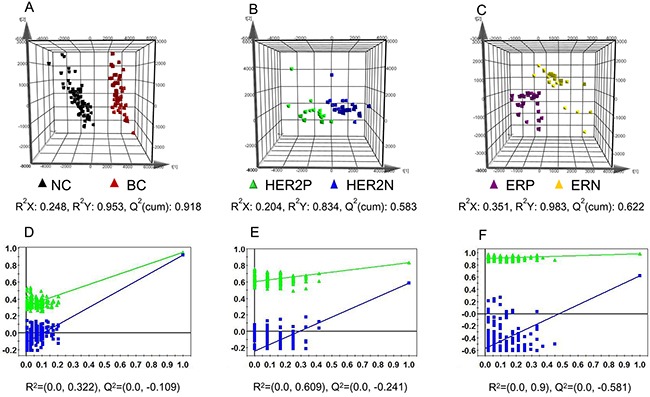
PLS-DA loading plots and chance permutation test obtained from LC-MS in positive mode **A.** Normal control (NC) *vs* breast cancer (BC) group; **B.** HER2-positive (HER2P) *vs* HER2-negative (HER2N) BC group; **C.** ER-positive (ERP) *vs* ER-negative (ERN) group. Black triangle corresponds to NC group, red triangle corresponds to BC group, green triangle corresponds to HER2-positive BC patients, blue triangle corresponds to HER2-negative BC subjects, purple triangle corresponds to ER-positive participants, and yellow triangle corresponds to ER-negative patients. Chance permutation at 200 times was used for the discrimination between **D.** NC *vs* BC, **E.** HER2P *vs* HER2N, and **F.** ERP *vs* ERN.

Clinical characteristics of BC patients and NC subjects were summarized in Table 1. Detailed patient information, stages of disease and other parameters were shown in Supplementary Table S1 and Table S2. Typical immunohistochemical pathology of different receptor statuses in accordance with the FDA-approved system was provided in Supplementary Figure S1. Typical total ion chromatograms (TICs) of a BC sample obtained from ESI^+^, ESI^−^, and GC-Q/MS were provided in Supplementary Figure S2. As shown in Figure 1, clear discriminations were obtained by ESI^+^ between BC and NC groups (Figure 1A), HER2-positive and HER2-negative BC groups (Figure 1B), ER-positive and ER-negative BC groups (Figure 1C). Similar discriminations were also observed by ESI^−^ (Supplementary Figure S3) and GC-Q/MS (Supplementary Figure S4). The metabolites with variable importance in the project (VIP) higher than 1 in loading plot were highlighted as biomarker candidates (Supplementary Figure S5). Additionally, Student's *t* test was used to validate the significance of the difference in intensities between variables.

**Table 1:** Clinical characteristics of the patients with breast cancer

Samples		HER2 positive (IHC + + + and + + with gene amplification by FISH)	HER2 negative (IHC−, +, and ++ without gene amplification by FISH)	Normal control	*p*
**Sample No.**		36	60	79	
**Age(year)**		52.3(31~82)	53.1(31~78)	46.4(24~86)	>0.05
**ER positive**		*n*=11 (5 for test set) as Luminal B subtype	*n*=42 (22 for test set) as Luminal A subtype	\	
**ER negative**		*n*=25 (10 for test set) as HER2-enriched subtype	*n*=18 (8 for test set) as Basal-like subtype	\	
**TNM classification stage**	I	6	25	\	
	IIA	19	20	\	
	IIIA	1	1	\	
	IIIB	10	14	\	

HER2, human epidermal growth factor receptor 2;

ER, estrogen receptor;

IHC, immunohistochemistry;

FISH, fluorescence *in situ* hybridization.

### Discrimination of BC and NC groups

A total of 1957 peaks were detected from ESI^+^ LC-MS, 1329 peaks from ESI^−^ LC-MS, and 123 peaks from GC-MS. The significant ions were then imported into the SIMCA-P 11.5 software package. Figure 1A illustrated score plots of the partial least squares discriminant analysis (PLS-DA) model of BC patients and NC participants. In Figure 1A, BC patients were clearly separated from NC group. The cumulative R^2^Y and Q^2^ were 0.953 and 0.918. The chance permutations at 200 times produced R^2^Y-intercept and Q^2^-intercept at 0.322 and −0.109 (Figure 1D), indicating that no over-fitting was observed.

**Figure 2:**
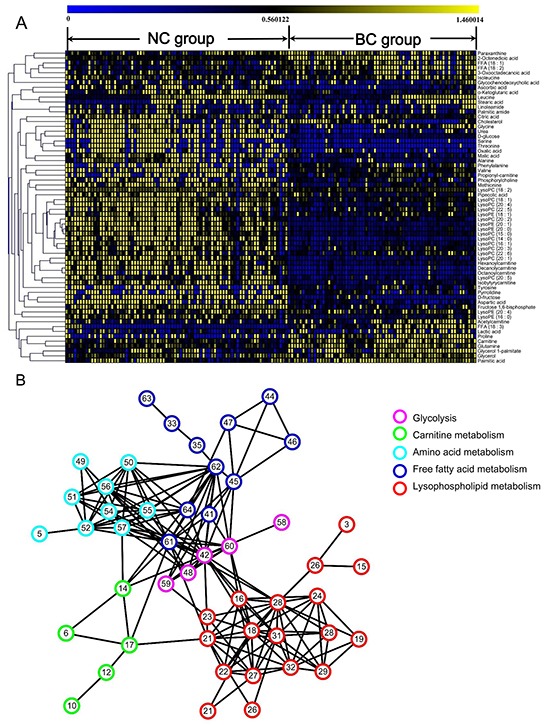
The identified differential metabolites between normal control (NC) and breast cancer (BC) groups **A.** Heatmap of 64 differential metabolites between BC and NC participants. The colors from blue to yellow indicate the elevated amount of metabolites. **B.** Correlation network analysis of differential metabolites. Metabolites with high correlation coefficients were connected by lines.

Sixty-four significantly altered plasma metabolites in BC patients relative to NC group were identified from the two-component PLS-DA model, in which 32 were further confirmed using reference compounds. The differential metabolites and their pathways were presented in Supplementary Table S3. Their relative normalized quantities were plotted in a heat map in Figure 2A.

### Correlation network of differential metabolites in BC plasma

A correlation network analysis was established using Cytoscape software in Figure 2B. Highly correlated metabolites were connected with a line. Glycolysis-related metabolites were located in the center of the network with an elevated tendency. A positive correlation was observed between the levels of glycolysis-related metabolites and fatty acids, indicating the high energy consumption from aerobic glycolysis during fatty acid biosynthesis in cancer. Lipids, especially lysophospholipids, exhibited a significantly decreased amount in the network. For most of the amino acids, there was a negatively correlated regulation.

### Discrimination of HER2-positive and HER2-negative BC

**Figure 3:**
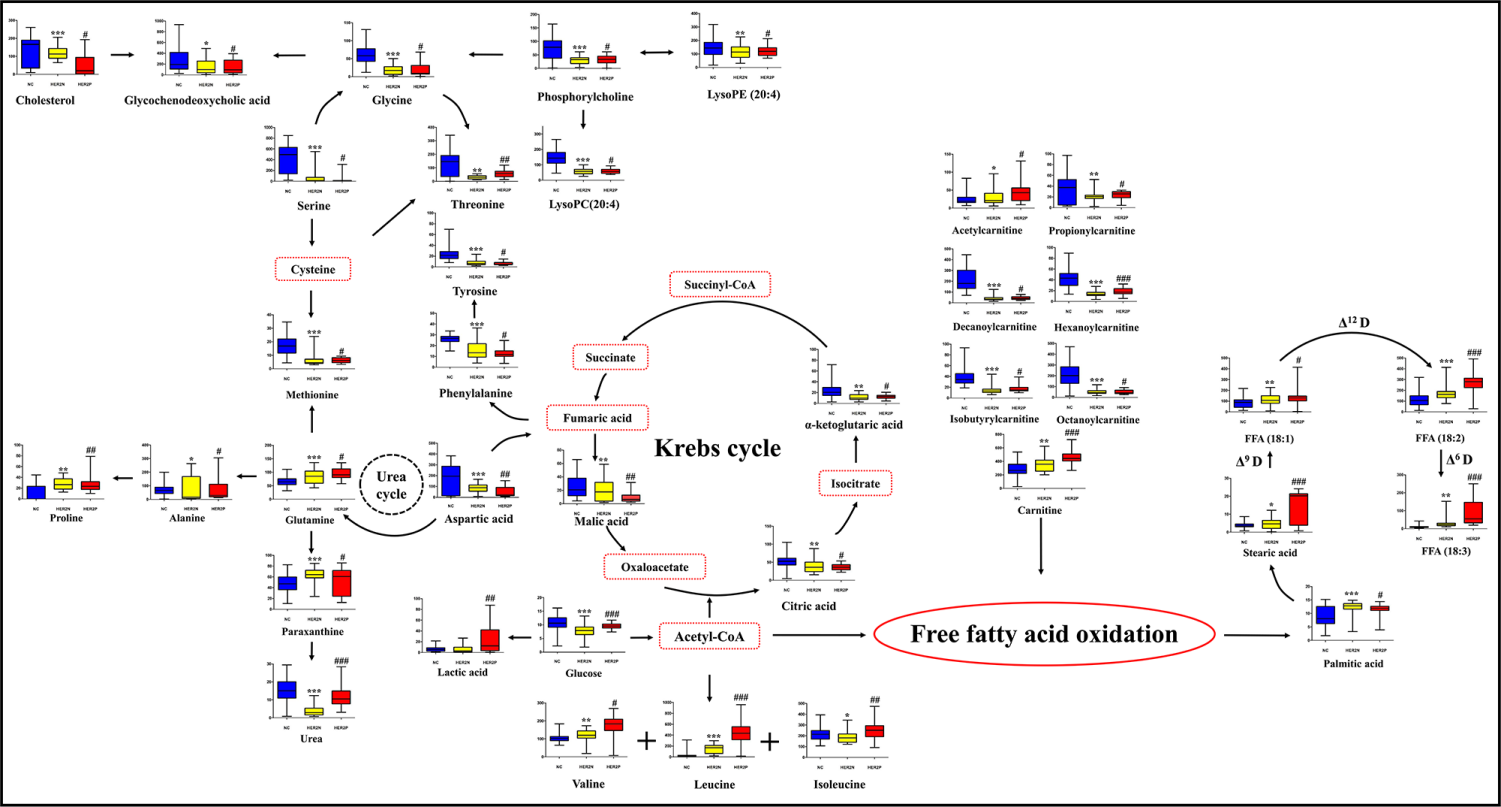
HER2-related altered metabolic pathway network of the significantly regulated metabolites Blue, yellow, and red charts represent the relative intensities of differential metabolites in normal control, HER2-negative, and HER2-positive breast cancer groups. The names with dashed red lines represent the undetected metabolites. The names with full red lines represent the detected metabolic reactions. LysoPC, lysophosphorylcholine; LysoPE, lysophosphoethanolamine; FFA, free fatty acid; Δ^6^ D, Δ^6^ desaturase. Acetyl-CoA, acetyl-coenzyme A; Succinyl-CoA, succinyl-coenzyme A. * represents the difference between HER2-negative breast cancer group and normal subjects, *, *p* < 0.05; **, *p* < 0.01; ***, *p* < 0.001, # represents the difference between HER2-positive patients and HER2-negative participants, #, *p* < 0.05; ##, *p* < 0.01; ###, *p* < 0.001.

As shown in Figure 1B, we demonstrated significant differences between the HER2-positive patients and HER2-negative BC subjects in the PLS-DA score plot. Permutation results were shown in Figure 1E. Using a combination of the VIP > 1 from PLS-DA with results from Student's *t* test, 40 metabolites (Table 2) were identified as differential variables. Heatmap of 40 differential metabolites were shown in Supplementary Figure S6. HER2-related altered metabolic pathway network of the significantly regulated metabolites was provided in Figure 3.

**Table 2:** Differential metabolites identified between HER2 positive breast cancer and HER2 negative breast cancer and their pathway involved

No.	*t_R_*(min)	*m/z*	Metabolites	Formula	Fold change ^*a*^	*p* value	VIP ^*b*^	Pathway involved
**ESI^+^**								
1	0.676	147.0604	Glutamine^*^	C_5_H_10_N_2_O_3_	1.287	<0.001	1.232	Alanine, aspartate and glutamate metabolism
2	0.733	162.1124	Carnitine^*^	C_7_H_15_NO_3_	1.367	<0.001	2.721	Fatty acid transportation
3	0.743	116.0711	Proline^*^	C_5_H_9_NO_2_	1.217	0.007	2.771	Arginine and proline metabolism
4	0.959	118.0869	Valine^*^	C_5_H_11_NO_2_	1.187	0.002	2.749	Valine, leucine and isoleucine metabolism
5	1.015	204.1231	Acetylcarnitine^*^	C_9_H_17_NO_4_	1.666	0.003	2.337	Fatty acid transportation
6	1.018	130.0861	Pipecolic acid	C_6_H_11_NO_2_	0.746	0.024	1.485	Protein synthesis, amino acid biosynthesis
7	1.242	150.0548	Methionine^*^	C_5_H_11_NO_2_S	1.260	<0.001	1.986	Cysteine and methionine metabolism
8	1.468	182.0815	Tyrosine^*^	C_9_H_11_NO_3_	1.227	0.030	1.036	Aminoacyl-tRNA biosynthesis
9	1.638	218.1378	*Propionyl-carnitine*	C_10_H_19_NO_4_	1.238	<0.001	1.622	Fatty acid transportation
10	1.751	166.0871	Phenylalanine^*^	C_9_H_11_NO_2_	1.375	<0.001	2.221	Aminoacyl-tRNA biosynthesis
11	1.977	232.1545	Isobutyryl-carnitine	C_11_H_21_NO_4_	1.334	0.011	1.454	Fatty acid transportation
12	2.712	260.1855	Hexanoylcarnitine	C_13_H_25_NO_4_	1.260	0.010	1.453	Fatty acid transportation
13	3.448	288.2172	*Octanoylcarnitine*	C_15_H_29_NO_4_	1.254	0.019	1.839	Fatty acid transportation
14	4.240	502.2933	*LysoPE (20:4)*	C_25_H_44_NO_7_P	1.497	0.001	2.274	Lysophospholipid catabolism
15	4.409	316.2485	*Decanoyl-L-carnitine*	C_17_H_33_NO_4_	1.386	0.004	1.626	Fatty acid transportation
16	4.636	544.3397	LysoPC(20:4)^*^	C_28_H_50_NO_7_P	1.299	0.020	2.653	Glycerophospholipid catabolism
17	4.749	184.0734	Phosphorylcholine	C_5_H_14_NO_4_P	0.721	0.007	1.009	Glycerophospholipid catabolism
18	4.862	480.3079	*LysoPE(18:1)*	C_23_H_46_NO_7_P	1.484	0.003	2.083	Lysophospholipid catabolism
19	5.654	522.3550	*LysoPC (18:1)*	C_26_H_52_NO_7_P	1.287	0.003	2.251	Glycerophospholipid catabolism
**ESI^−^**								
20	0.688	179.0579	Paraxanthine^*^	C_7_H_8_N_4_O_2_	1.129	0.004	1.141	Purine metabolism
21	0.907	145.0139	α-Ketoglutaric acid	C_5_H_6_O_5_	0.713	0.038	1.101	Tricarboxylic acid cycle
22	1.423	133.0139	Malic acid^*^	C_4_H_6_O_5_	0.461	0.004	1.571	Tricarboxylic acid cycle
23	1.536	130.0871	Isoleucine^*^	C_6_H_13_NO_2_	1.545	<0.001	1.957	Valine, leucine and isoleucine metabolism
24	2.611	171.0659	*2-Octenedioic acid*	C_8_H_12_O_4_	1.234	<0.001	2.565	Fatty acid metabolism
25	3.573	448.3075	Glycochenodeoxycholic acid^*^	C_26_H_43_NO_5_	0.566	0.026	1.980	Bile acid biosynthesis
26	6.684	277.2174	FFA (18:3)^*^	C_18_H_30_O_2_	1.422	0.007	1.746	Biosynthesis of unsaturated fatty acids
27	7.532	279.2322	FFA (18:2)^*^	C_18_H_32_O_2_	1.461	<0.001	1.864	Biosynthesis of unsaturated fatty acids
28	8.833	281.2486	FFA (18:1)^*^	C_18_H_34_O_2_	1.174	0.013	1.416	Biosynthesis of unsaturated fatty acids
**GC-MS**								
29	6.299		Lactic acid^*^	C_3_H_6_O_3_	1.443	<0.001	1.208	Glycolysis metabolism
30	6.904		Alanine	C_3_H_7_NO_2_	1.023	<0.001	1.190	Alanine and aspartate metabolism
31	7.117		Glycine^*^	C_2_H_5_NO_2_	2.252	<0.001	1.961	Glycine, serine and threonine metabolism
32	8.879		Urea	CH_4_N_2_O	2.574	0.012	1.455	Urea cycle
33	10.612		Serine^*^	C_3_H_7_NO_3_	2.090	<0.001	1.084	Glycine, serine and threonine metabolism
34	10.957		Threonine^*^	C_4_H_9_NO_3_	2.748	<0.001	1.028	Glycine, serine and threonine metabolism
35	11.871		Aspartic acid^*^	C_4_H_7_NO_4_	0.559	<0.001	1.162	Alanine, aspartate and glutamate metabolism
36	16.230		Citric acid^*^	C_6_H_8_O_7_	0.837	<0.001	1.856	Tricarboxylic acid cycle
37	17.034		D-glucose^*^	C_6_H_12_O_6_	0.333	<0.001	1.794	Glycolysis metabolism
38	18.045		Palmitic acid^*^	C_16_H_32_O_2_	1.789	<0.001	1.739	Fatty acid biosynthesis
39	19.862		Stearic acid^*^	C_18_H_36_O_2_	1.223	0.002	1.017	Fatty acid biosynthesis
40	27.843		Cholesterol^*^	C_27_H_46_O	0.832	<0.001	2.359	Hormone biosynthesis and bile acid biosynthesis

* confirmed with reference standards;

a fold change >1 indicates that the average normalized peak area ratio in HER2-positive group is larger than that in HER2-negative group;

b variable importance in the projection.

Metabolites in italic were variables with VIP>1.5.

### Discrimination of ER-positive and ER-negative BC

**Figure 4:**
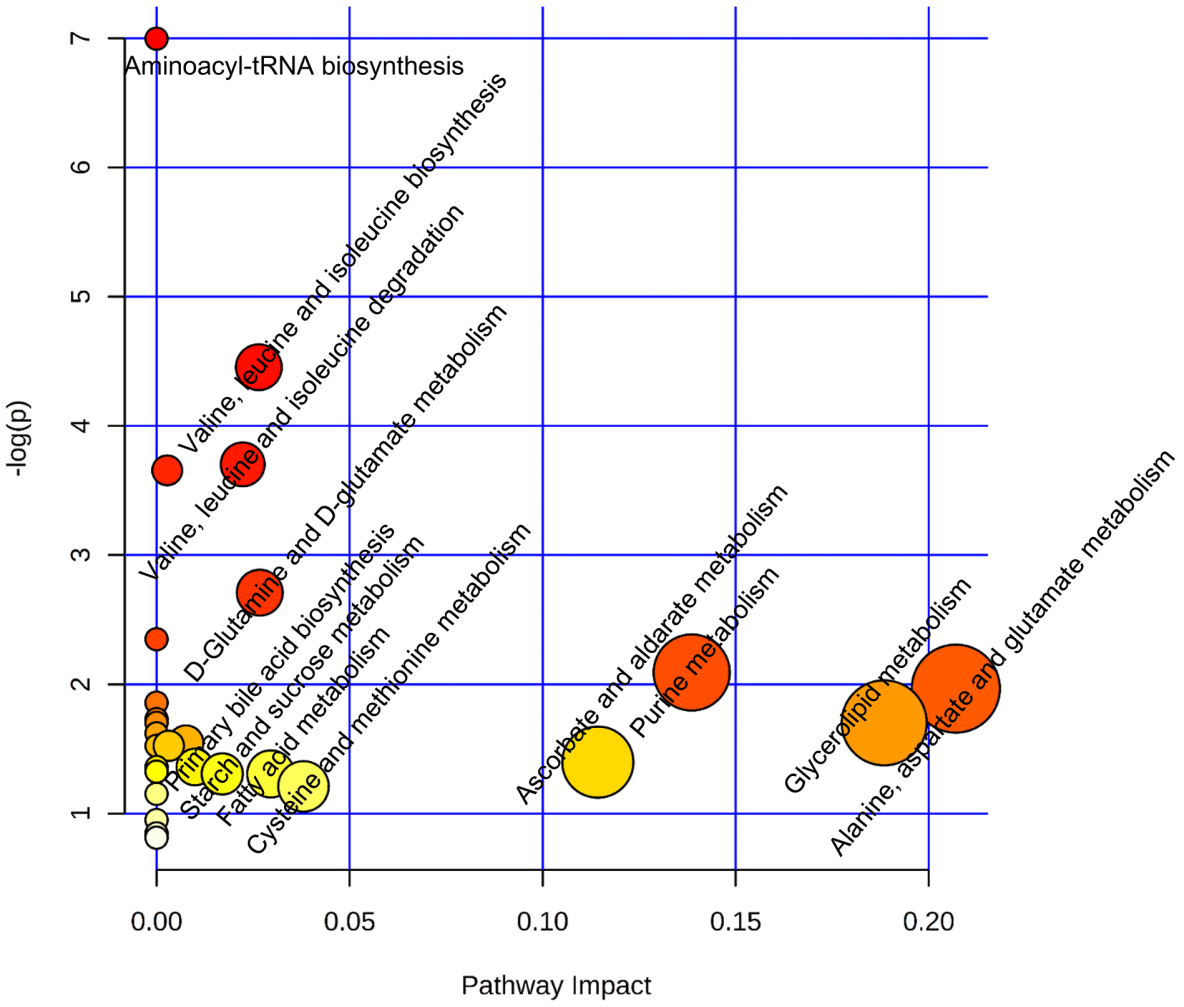
Disturbed metabolic pathways in ER-positive compared with ER-negative BC Metaboanalyst (*http://www.metaboanalyst.ca*) generated topology map described the impact of baseline metabolites identified between ER-positive *vs* ER-negative groups with high VIP values (VIP>1) on metabolic pathways.

As shown in Figure 1C, significant differences were observed between the ER-positive and ER-negative patients. Table 3 listed the 22 differential metabolites identified with the VIP > 1 from PLS-DA and results from Student's *t* test. ER-related disturbed metabolic pathways were shown in Figure 4.

**Table 3:** Differential metabolites identified between ER positive breast cancer and ER negative plasma and their pathway involved

No.	*t_R_*(min)	*m/z*	Metabolites	Formula	Fold change ^*a*^	*p* value	VIP^*b*^	Pathway involved
**ESI^+^**								
1	0.676	147.0604	Glutamine^*^	C_5_H_10_N_2_O_3_	1.159	0.019	1.650	Alanine, aspartate and glutamate metabolism
2	0.733	162.1124	Carnitine^*^	C_7_H_15_NO_3_	1.187	0.006	2.373	Fatty acid transportation
3	0.959	118.0869	Valine^*^	C_5_H_11_NO_2_	0.682	<0.001	2.813	Valine, leucine and isoleucine metabolism
4	1.242	150.0548	Methionine^*^	C_5_H_11_NO_2_S	1.284	0.025	1.942	Cysteine and methionine metabolism
5	4.296	468.3086	*LysoPC(14:0)*	C_22_H_46_NO_7_P	0.751	0.001	2.257	Glycerophospholipid catabolism
6	4.466	494.3242	LysoPC(16:1)^*^	C_24_H_48_NO_7_P	1.268	0.002	2.748	Glycerophospholipid catabolism
7	4.692	454.2957	LysoPE (16:0)	C_21_H_44_NO_7_P	1.227	0.027	1.111	Lysophospholipid catabolism
8	4.805	542.3224	*LysoPC (20:5)*	C_28_H_48_NO_7_P	0.649	0.037	2.358	Glycerophospholipid catabolism
9	4.805	482.3233	*LysoPC(15:0)*	C_23_H_48_NO_7_P	0.870	0.003	2.152	Glycerophospholipid catabolism
10	4.862	570.3547	LysoPC(22:5)	C_30_H_52_NO_7_P	0.799	<0.001	1.076	Glycerophospholipid catabolism
11	5.145	546.3559	*LysoPC(20:3)*	C_28_H_52_NO_7_P	0.794	0.006	1.701	Glycerophospholipid catabolism
**ESI^−^**								
12	0.688	179.0579	Paraxanthine^*^	C_7_H_8_N_4_O_2_	1.169	0.003	1.445	Purine metabolism
13	0.914	175.0248	Ascorbic acid^*^	C_6_H_8_O_6_	0.750	0.014	1.330	Ascorbic acid
14	1.423	133.0139	Malic acid^*^	C_4_H_6_O_5_	0.464	<0.001	1.373	Tricarboxylic acid cycle
15	1.536	130.0871	Isoleucine^*^	C_6_H_13_NO_2_	0.875	0.002	1.209	Valine, leucine and isoleucine metabolism
16	2.611	171.0659	*2-Octenedioic acid*	C_8_H_12_O_4_	1.104	<0.001	2.511	Fatty acid metabolism
17	3.573	448.3075	Glycochenodeoxycholic acid^*^	C_26_H_43_NO_5_	1.265	0.002	3.035	Bile acid biosynthesis
**GC-MS**								
18	6.904		Alanine^*^	C_3_H_7_NO_2_	2.056	<0.001	2.891	Alanine and aspartate metabolism
19	9.304		Leucine^*^	C_6_H_13_NO_2_	0.452	<0.001	1.085	Valine, leucine and isoleucine metabolism
20	9.403		Glycerol^*^	C_3_H_8_O_3_	1.069	0.004	1.476	Glycerophospholipid metabolism
21	17.034		D-glucose^*^	C_6_H_12_O_6_	1.218	0.003	2.193	Glycolysis metabolism
22	18.045		Palmitic acid^*^	C_16_H_32_O_2_	0.556	<0.001	1.900	Fatty acid biosynthesis

* confirmed with reference standards;

a fold change >1 indicates that the average normalized peak area ratio in ER-positive group is larger than that in ER-negative group;

b variable importance in the projection.

Metabolites in italic were variables with VIP>1.5.

### Diagnostic potential of differential metabolites for subtype classifications

**Figure 5:**
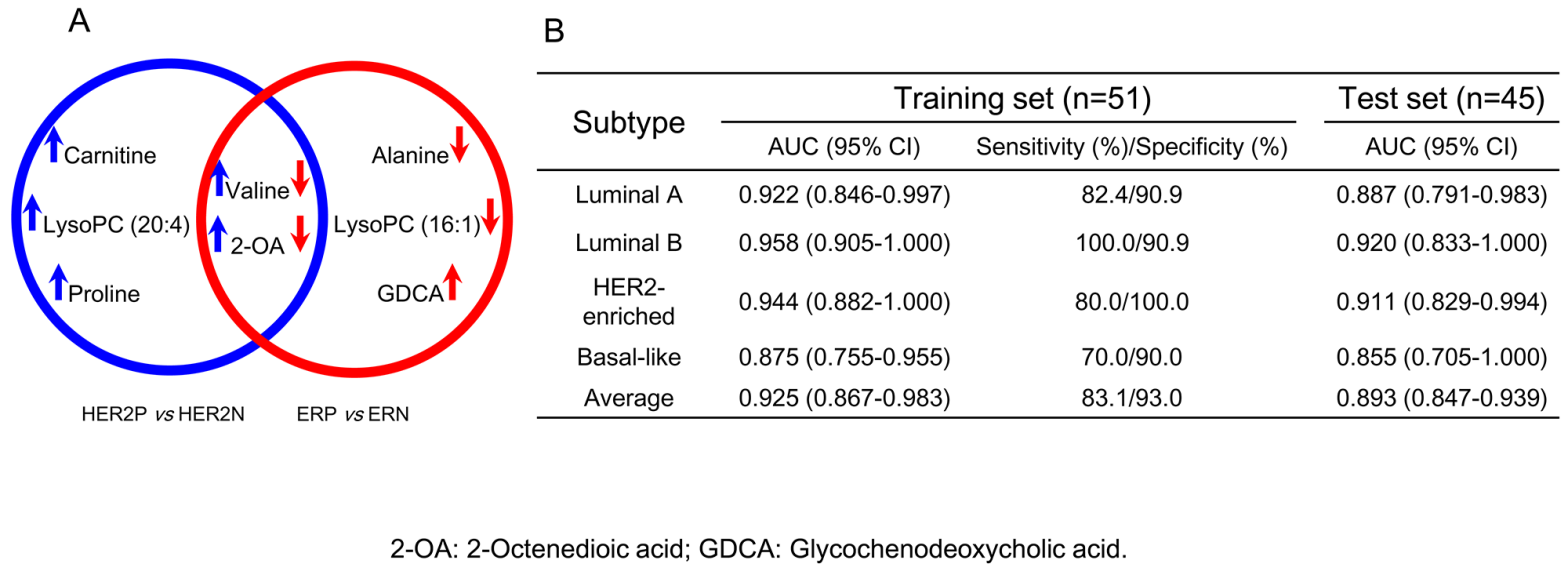
Combinational panel of 8 biomarkers and their diagnostic outcomes **A.** Venn diagram of the differential metabolites panels generated from the discrimination of different HER2 and ER statuses in breast cancer. The upward arrow represents an increased level of metabolite with the overexpression of HER2 (blue arrow) and ER (red arrow). LysoPC: lysophosphatidylcholine; 2-OA: 2-octenedioic acid; GDCA: glycochenodeoxycholic acid. **B.** Areas under the curve provided by the 8 biomarkers for the discrimination of BC subtypes in the training set and test set.

**Figure 6:**
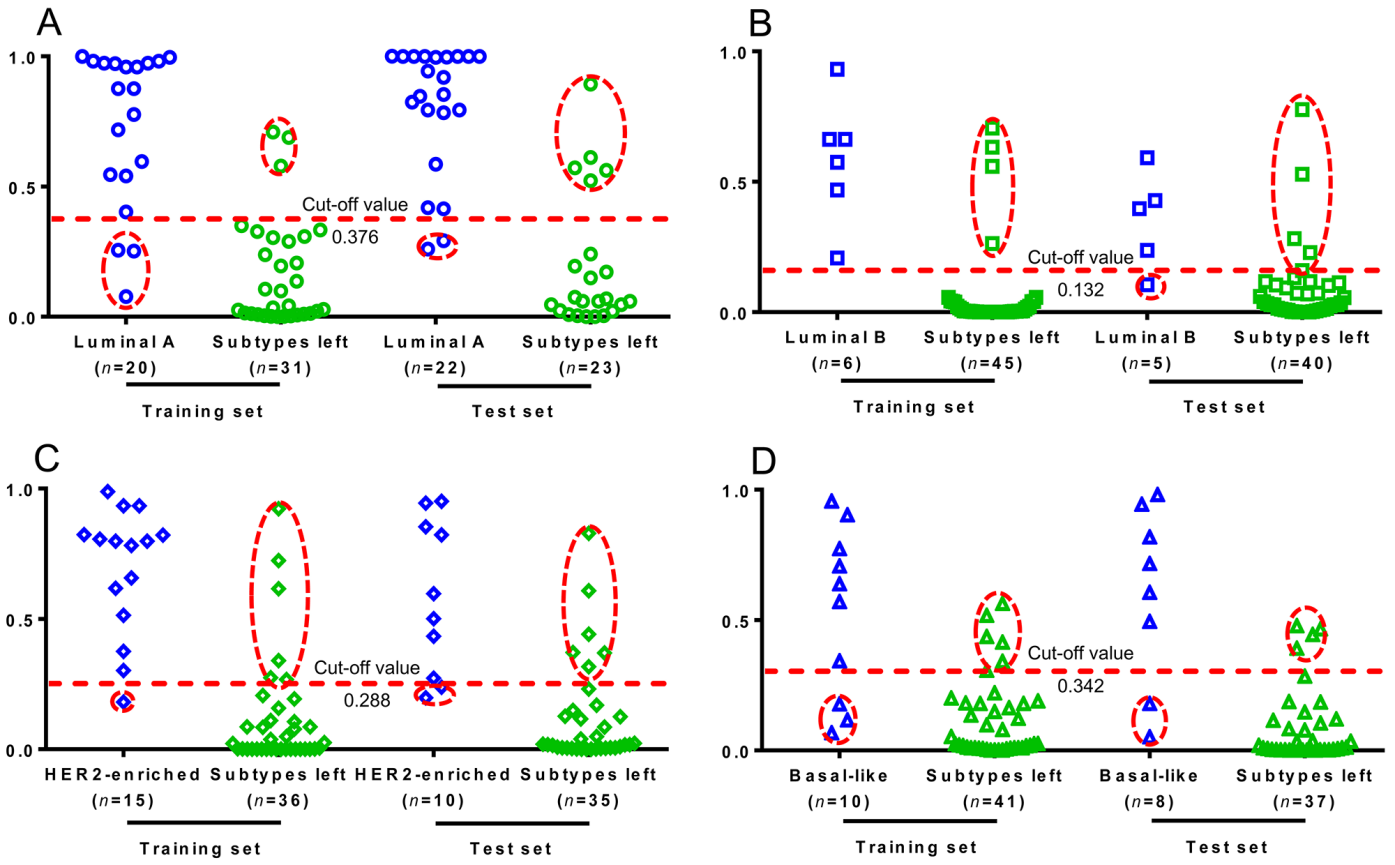
Prediction accuracies for BC subtypes based on the eight biomarkers The prediction plots based on the optimal cut-off value for **A.** Luminal A, **B.** Luminal B, **C.** HER2-enriched, and **D.** Basal-like BC subtypes. Plot in hollow dashed circle represents samples with false prediction.

The HER2 and ER statuses are the key to the classification of BC subtypes. The metabolites with VIPs > 2.5 responsible for the discrimination of HER2 in Table 2 and ER statuses in Table 3 were selected for potential diagnosis. A combinational panel of 8 metabolites was assigned as candidate markers shown in Figure 5A, including carnitine, lysophosphatidylcholines (lysoPC) (20:4), proline, alanine, lysoPC (16:1), glycochenodeoxycholic acid (GDCA), valine, and 2-octenedioic acid (2-OA). The performances of these 8 metabolites in the diagnosis of four clinical BC subtypes were conducted by ROC analysis. As shown in Figure 5B, the panel of 8 metabolites provided diagnostic abilities with average area under the curve at 0.925 (95% CI 0.867-0.983) for the training set (*n*=51) and 0.893 (95% CI 0.847-0.939) for the test set (*n*=45). Based on the highest prediction sensitivity and specificity of the ROC on the training set, we calculated the optimal cut-off values at 0.376 for Luminal A, 0.132 for Luminal B, 0.288 for HER2-enriched, and 0.342 for Basal-like subtypes (Figure 6). Using the optimal cut-off values, prediction accuracies in Figure 6A showed 88.3% for Luminal A subjects in the training set and 84.4% in the test set. Predictive accuracies in Figure 6B showed 92.2% for Luminal B patients in the training set and 88.9% for the test set. As shown in Figure 6C, predictive accuracies at 89.3% for HER2-enriched group in training set and 82.2% in the test set was obtained. In Figure 6D, we observed predictive accuracies at 84.3% for basal-like participants in the training set and 86.7% in the test set.

## DISCUSSION

This study describes for the first time the plasma metabolic profiling change specifically associated with BC subtypes. Metabolic phenotypes revealed significant pattern differences between BC and NC groups, HER2-positive and HER2-negative BC groups, ER-positive and ER-negative BC groups. In these datasets, there were few misclassifications by unbiased analysis. Progesterone receptor (PR) status should be considered in classification of breast cancer. Expressional consistency of ER and PR was observed in all samples.

The parallel use of LC-MS and GC-MS provided comprehensive distinct metabolites. We observed 64 most significantly regulated plasma metabolites between BC patients and NC group. They were classified as amino acids, free fatty acids (FFA), lysoPCs, lysophosphatidylethanolamines (lysoPEs), carnitines, and organic acids.

Comparison between HER2-positive and HER2-negative BC patients generated 40 differential metabolites. The principal metabolic changes in HER2-positive BC compared with HER2-negative BC included elevated aerobic glycolysis, enhanced gluconeogenesis, and increased fatty acid biosynthesis with reduced Krebs cycle and Δ^9^ desaturase. The elevated level of lactic acid and decreased D-glucose in plasma of HER2-positive BC characterized the strong aerobic glycolysis (Warburg effect) in cancer cells [15]. Gluconeogenesis in HER2-positive BC was upregulated for energy supply, resulting in enriched consumption of amino acids in gluconeogenesis [16]. A significant enrichment in unsaturated fatty acids (UFAs) was found in HER2-positive BC, implying the increased UFAs probably resulted from the *de nevo* biosynthesis of fatty acids and enhanced Δ-dehydrogenase during the cell proliferation and metastasis of HER2-positive BC [17].

The ER statuses in BC were considered. The present data suggested that the major altered pathways in ER-positive BC patients included elevated alanine, aspartate and glutamate metabolism, decreased glycerolipid catabolism, and enhanced purine metabolism, when compared with ER-negative group (Figure 4). Similar to HER2-positive BC group, lysoPCs were at low levels in ER-positive patients, corresponding to the strong negative correlation between cPLA2α mRNA expression and ER expression levels [18]. Elevated level of glutamine in ER-positive patients compared to ER-negative participants clearly point to the perturbation of glutamate-to-glutamine ratio. This result is in agreement with previous observations [19].

We identified a panel of 8 potential small-molecule biomarkers for the diagnosis of BC subtypes. Carnitine, as an essential for the entry of fatty acid into the mitochondria for β-oxidation [20], was observed at a high level (FC=1.367, *P*<0.001) in HER2-positive group, which might lead to the activated metabolism of fats. LysoPC (20:4), metabolic products of PC by hydrolysis of phospholipase A2 [18], were at a high level (FC=1.299, *P*=0.020) in HER2-positive patients. The results corresponded to an increased expression of cytosolic phospholipase A2-α in HER2 over-expression BC cell lines [21]. The elevated amount of proline (FC=1.217, *P*=0.007) might indicate a suppressed proline oxidase in HER2-positive group [22]. Alanine was the most significantly decreased metabolite (FC=0.544, *P*<0.001) in ER-positive participants compared with ER-negative group [23]. The reduced lysoPC (16:1) (FC=0.786, *P*=0.002) in ER-positive patients showed relation with the activity inhibition of phospholipase A2 in MCF-7 BC cells [18]. The increased GDCA (FC=1.265, *P*=0.002) in ER-positive group was highly related to the enhanced proliferation of cancer cells, corresponding to its higher morbidity [24]. Valine and 2-OA were the co-markers in the discrimination of BC with different HER2 and ER expression levels. They are significantly increased in HER2-postive compared to HER2-negative but decreased remarkably in ER-positive compared to ER-negative groups. The abnormalities of valine suggested the disorder of energy supply in HER2-postive (FC=1.187, *P*=0.002) and ER-positive (FC=0.682, *P*<0.001) patients. The marked regulation of 2-octenedioic acid was an indicator for the abnormal fatty acid metabolism in HER2-positive (FC=1.234, *P*<0.001) and ER-positive (FC=0.833, *P*<0.001) subjects [25].

The clinical predictive potential of the identified 8 biomarkers was highlighted in this work for BC subtypes. Average predictive accuracies at 88.5% (95% CI 83.3%-93.7%) were obtained for the training set and 85.6% (95% CI 80.9%-90.1%) for the test set. We also used a panel of 29 metabolites with VIPs>1.5 (metabolites in italic in Tables 2 and 3) instead of 8 metabolites with VIPs>2.5 for prediction of breast cancer subtypes. The average predictive accuracies increased to 97.1% (95% CI 93.0%-100.0%) for training sets and 95.6% (95% CI 92.7%-98.5%) for test sets. In consideration of the clinical use of 29 metabolites is difficultly popularized due to the limited standards, 8 metabolites with VIPs>2.5 were more applicable as the diagnostic biomarkers.

In conclusion, this study is a first clinical metabolic research for BC subtype classification. We demonstrate a clear move toward discovering the metabolomic drivers for the various BC subtypes. We suggest that plasma metabolomic test is faster, less costly, and noninvasive, and could be used as a pre-screen to other forms of more invasive or uncomfortable screening. These metabolomic data can also help to identify new therapeutic pathways from which novel agents might be developed. In future, we will undertake study of a larger prospective cohort to further validate the accuracy of this test. An evaluation of the mechanisms of BC subtypes by general and targeted metabolomics as well as other systematic biological approaches could be used.

## MATERIALS AND METHODS

### Clinical sample collection

We collected plasma samples from 175 participants and all the subjects signed the informed consents before sample collection. In total, 96 BC patients, aged 31 to 82 years old, were enrolled in this work. This study was conducted with the guide of the Helsinki Declaration and the International Conference on Harmonization-Good Clinical Practices (ICH-GCP). This study was approved by the Institutional Review Boards of the First Affiliated Hospital of Nanjing Medical University, Jiangsu Province Hospital with approval number 2011-SRFA-058. The patients selecting protocol was set as follows: all the participants should sign the informed consent; patients diagnosed with BC should be confirmed by histology; patients should receive no surgical operation before this research; participants have sufficient heart, lung, liver, kidney, and hematopoietic functions with Eastern cooperative oncology group (ECOG) performance status ≤ 2, and weight loss < 10% in recent 6 months. Cancer stage was classified according to the 2002 Tumor Nodes Metastasis (TNM) staging system. Particularly, BC patients diagnosed with HER2 (−), HER2 (+), or HER2 (+ +) without gene amplification were defined as HER2-negative BC. Patients diagnosed with HER2 (+ + +) or HER2 (+ +) with gene amplification were classified into the HER2-positive BC group. NC samples were collected from a total of 79 healthy volunteers between the ages of 24 and 86 according to the same sample collection protocol. All the samples were randomly classified into training set and test set. Detailed baseline characteristics of patients enrolled in this study were provided in Table 1.

Fasting blood samples collected in the morning from all the subjects were stored in K2 EDTA vacutainer tubes and cooled down in freezer (4°C) at once. They were then centrifuged at 3000 × g for 10 min at 4°C within 2 h. Supernatants (plasma) were transferred into new vials, and immediately stored frozen (−80°C) until sample preparation. The sample pretreatment methods for LC-MS and GC-MS were detailed in the methods provided in Methods S1.

As part of the quality control (QC) and system conditioning process, a pooled QC sample was prepared by mixing equal volumes (10 μL) of the collected 175 samples.

### Statistical analysis

The acquired MS data from GC-Q/MS and UPLC-Q/TOF-MS in both positive and negative ion modes were imported into the SIMCA-P software (version 11.5, Umetrics) for multivariate analysis. GraphPad Prism 5 package was applied to plot the relative amount of each metabolite. Heatmaps and hierarchical cluster analysis (HCA) were conducted using the MeV software package (version 4.6.0), and the correlation network was established using the Cytoscape software package. ROC analysis and binary logistic regression were applied using SPSS version 19.

### Metabolites identification

GC-Q/MS metabolites were identified by comparing the mass fragmentations with NIST 05 Standard mass spectral databases in NIST MS search 2.0 (NIST, Gaithersburg, MD) software with a similarity of more than 70% and finally verified by available reference standards. Differential metabolites obtained from positive and negative ion modes of UPLC-Q/TOF-MS analyses were identified with available reference standards in our lab and the web-based resources such as the Human Metabolome Database (http://www.hmdb.ca/) and METLIN (http://metlin.scripps.edu/index.php) data source.

### Metabolomics pathway analysis

Database sources, including the KEGG (http://www.genome.jp/kegg/), MetaboAnalyst (http://www.metaboanalyst.ca/MetaboAnalyst/), Human Metabolome Database, and METLIN, were used for the identification of affected metabolic pathways.

## SUPPLEMENTARY FIGURES AND TABLES


