# Supplementary material for: Human plasma metabolomics for identifying differential metabolites and predicting molecular subtypes of breast cancer
Source: Oncotarget. 2016 Feb 3;7(9):9925–38. doi: 10.18632/oncotarget.7155 (PMC4891093; doi:10.18632/oncotarget.7155)
Supplement: Supplementary file 1 [file oncotarget-07-09925-s001.pdf]

SUPPLEMENTARY FIGURES AND TABLES

SUPPLEMENTARY MATERIALS AND METHODS

Chemicals and reagents

Acetonitrile and formic acid (UPLC grade) were purchased from Roe (Nowark, USA). Chromatographic methanol and chloroform was purchased from Hanbang Tech Co. (Jiangsu, China). Methoxyamine hydrochloride and N-methyl-N- trimethylsilyltrifluoroacetamide (MSTFA) with 1% trimethylchlorosilane (TMCS) for derivation was obtained from Sigma-Aldrich (St. Louis, MO). Deionized water (18 MΩ/cm) was further purified by a Milli-Q system (Millipore, Milford, MA, USA). Standard compounds, including 2-isopropylmalic acid, glutamine, proline, methionine, tyrosine, phenylalanine, lysoPC (20:4), lysoPC (16:1), paraxanthine, ascorbic acid, malic acid, isoleucine, glycochenodeoxycholic acid, FFA (18:1), FFA (18:2), FFA (18:3), lactic acid, glycine, oxalic acid, leucine, glycerol, serine, threonine, aspartic acid, citric acid, D-fructose, D-glucose, palmitic acid, stearic acid, and cholesterol were purchased from Sigma-Aldrich (St. Louis, MO).

Plasma sample preparation and analysis by GC-Q/MS

A 100 μL aliquot of plasma sample was spiked with internal standard (15 μL 2- isopropylmalic acid in water, 1.0 mg/mL) and vortexed for 10 s. The mixed solution was extracted with 300 μL of methanol / chloroform / water (2.5 : 1 : 1) and shaken at 1,200 rpm for 30 min at 37°C before being centrifuged at 16,000 × g for 5 min at 4°C. An aliquot of 450 μL of the supernatant obtained was transferred to a clean tube, and 400 μL of distilled water were added to the tube. After being mixed, the solution was centrifuged at 16,000 × g for 5 min at 4°C, and 500 μL of the supernatant was transferred to a glass sampling vial and dried under a gentle stream of nitrogen at room temperature. The residue derivation involved the addition of 80 μL methoxyamine hydrochloride (20 mg/mL in pyridine), was added to the vial and kept at 37°C for 16 h followed by the addition of 40 μL of MSTFA (1% TMCS) for 60 min at 70°C.

Each 0.2 μL aliquot of the derivatized solution was injected in spitless mode into an Agilent 7890B gas chromatography coupled with an Agilent 5977A mass spectrometer. Separation was achieved on a HP-5MS capillary column (30 m×0.25 mm I.D., 0.25-μm film thickness; Agilent J & W Scientific, Folsom, CA, USA) with helium as the carrier gas at a constant flow rate of 0.7 mL/min. The solvent delay time was set to 5 min. The temperature of injection, transfer interface, and ion source were set at 270°C, 290°C, and 230°C, respectively. The GC temperature programming was set to 2 min isothermal heating at 80°C, followed by 10°C/min oven temperature ramps to 300°C, and a final 6 min maintenance at 300°C. Electron impact ionization (70 eV) at full scan mode (m/z 30–600) was used.

Plasma sample preparation and analysis by UPLC-Q/TOF-MS

Fasting blood samples collected in the morning from all the subjects were stored in K2 EDTA vacutainer tubes and cooled down in freezer (4°C) at once. They were then centrifuged at 3000 × g for 10 min at 4°C within 2 h. Supernatants (plasma) were separated and transferred into new vials, and immediately stored frozen (-80°C) until sample preparation.

An aliquot of 150 μL of acetonitrile was added to 50 μL of plasma and the mixture was vortexed for 10 seconds. Then, at 4°C, the mixture was centrifuged for 10 minutes at a rotation speed of 13,000 rpm. After that, 150 μL of supernatant was transferred to a clean tube and dried under a gentle stream of nitrogen at room temperature. Finally, the dried supernatant was dissolved with 100 μL water/acetonitrile (4:1) solution and 1 μL of the sample injected for UPLC-Q/TOF-MS analysis.

The 1 μL aliquot of the supernatant was injected into a 100 mm × 2.1 mm, 1.7 μm Syncronis aQ C18 column (Thermo, USA) held at 45°C using an Agilent 1290 infinity system (Agilent technologies, Germany). Mobile phases consisted of water with 0.1% formic acid (A) and acetonitrile with 0.1% formic acid (B). The column was eluted with a linear gradient of 1-20% B over 0-1 min, 20-70% B over 1-3 min, 70-85% B over 3-8 min, 85-100% B over 8-9 min, the composition was held at 100% B for 1 min. The flow rate was set at 0.4 mL/min.

Mass spectrometry was performed on an Agilent 6530 Q-TOF/MS system (Agilent Technologies, USA) equipped with an electrospray ionization (ESI) source. The conditions of the ESI source were as follows: drying gas (N2) flow rate, 10.0 l/min; drying gas temperature, 320°C; nebulizer pressure, 35 psig; capillary voltage, 3500 V; fragmentor voltage, 120 V. The analysis was performed using full scan mode, and the mass range was set at 50-1000 Da.

Samples from NC participants and BC patients were alternated in random order in the analysis batch. Before analyzing the sample sequence, five QC samples were run. To visually evaluate chromatographic reproducibility, during analysis of the sample sequence, the QC sample was analyzed repeatedly within the analytical run after every ten plasma samples.

Data processing methods

The acquired MS data (.d) from GC-MS and UPLC-Q/TOF-MS were exported to common data format (.mzdata) files by MassHunter Workstation Software (Version B.06.00, Agilent Technologies). The program XCMS (http://metlin.scripps.edu/download/) was applied for data pretreatment procedures such as nonlinear retention time alignment, peak discrimination, filtering, alignment, matching, and identification. peak alignment of the data in the time domain and automatic integration and extraction of the peak intensities. The 80% rule was used to treat the missing values for each sample group, then a list of the intensities of each peak detected was generated, using retention time and the *m/z* data pairs as the parameters for each ion.

The partial least-squared discriminant analysis (PLS-DA) were used to identify the variables responsible for the discrimination. Variable importance in the projection (VIP) analysis was applied to obtain the significant variables for subsequent metabolic pathway analysis. We calculated the R^2Y (cum) to estimate the "goodness of fit" of the model, and Q^2 (cum) to estimate the ability of prediction.

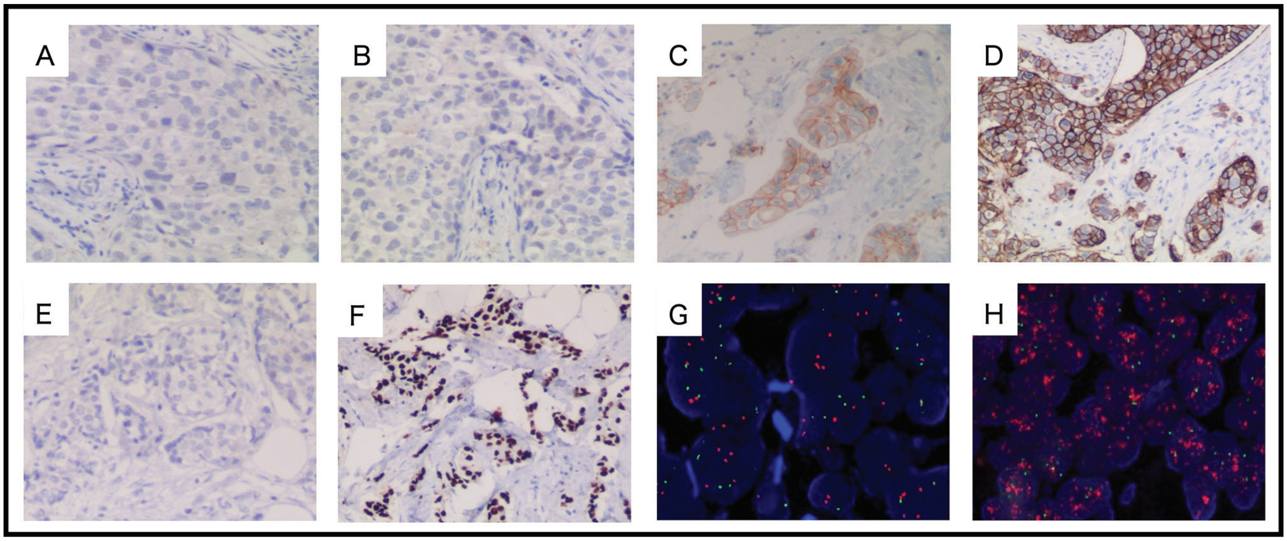

Figure S1: Pathology of HER2 and ER status in breast cancer patients. typical immunohistochemical pathology of **A.** HER2 (−), **B.** HER2 (+), **C.** HER2 (+ +), **D.** HER2 (+ + +), **E.** ER-negative, and **F.** ER-positive breast cancer patients; typical fluorescence *in situ* hybridization with the ratio of HER2 signals to CEP17 signals < 2.0 considered as non-amplified **G.** and those ≥ 2.0 considered as amplified **H.**

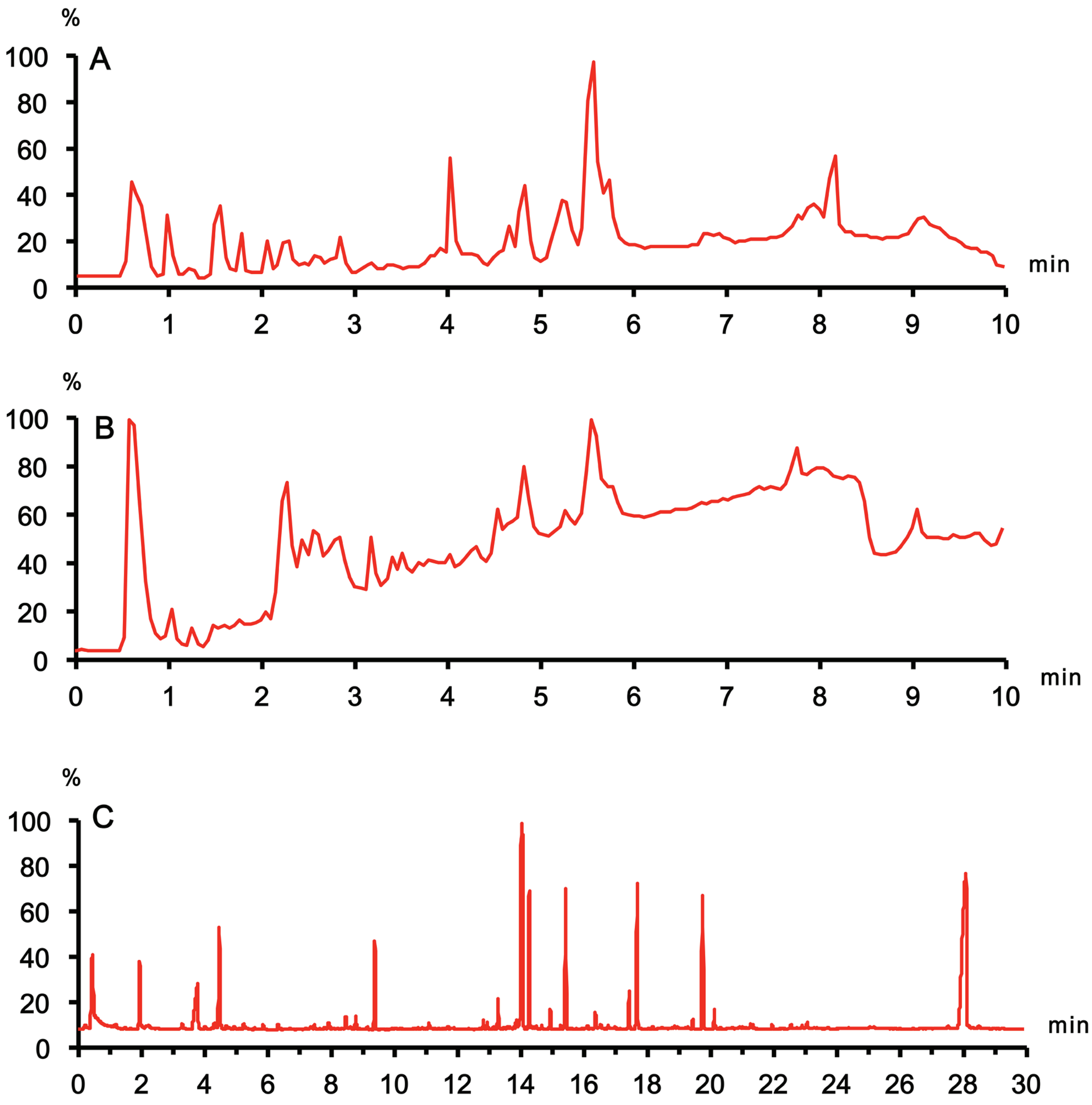

Figure S2: Typical total ion chromatograms (TICs) obtained from A. ESIfi, **B.** ESIfi, and **C.** GC-Q/MS.

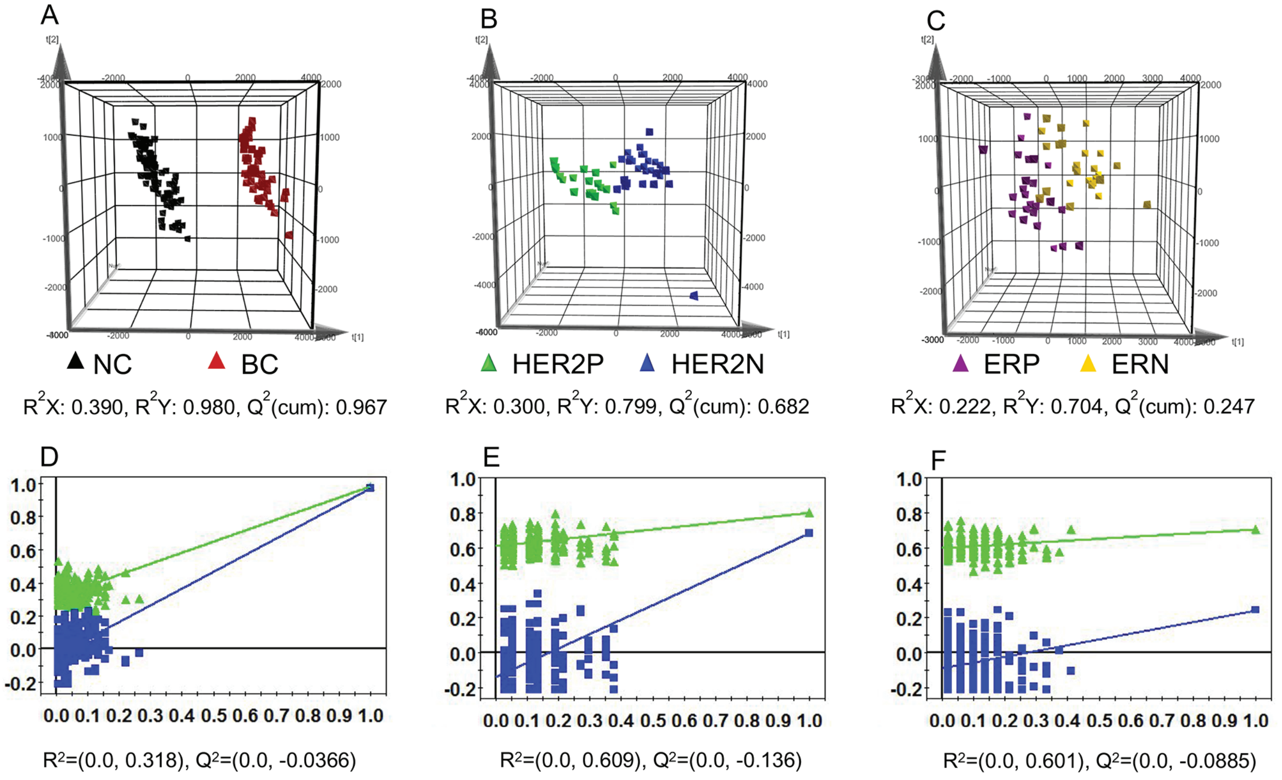

Figure S3: PLS-DA loading plots and chance permutation test obtained from LC-MS in negative mode. A. Normal control (NC) vs breast cancer (BC) group; **B.** HER2-positive (HER2P) vs HER2-negative (HER2N) BC group; **C.** ER-positive (ERP) vs ER-negative (ERN) group. Black triangle corresponds to NC group, red triangle corresponds to BC group, green triangle corresponds to HER2-positive BC patients, blue triangle corresponds to HER2-negative BC subjects, purple triangle corresponds to ER-positive participants, and yellow triangle corresponds to ER-negative patients. Chance permutation at 200 times for the discrimination between **D.** NC vs BC, **E.** HER2P vs HER2N, **F.** ERP vs ERN.

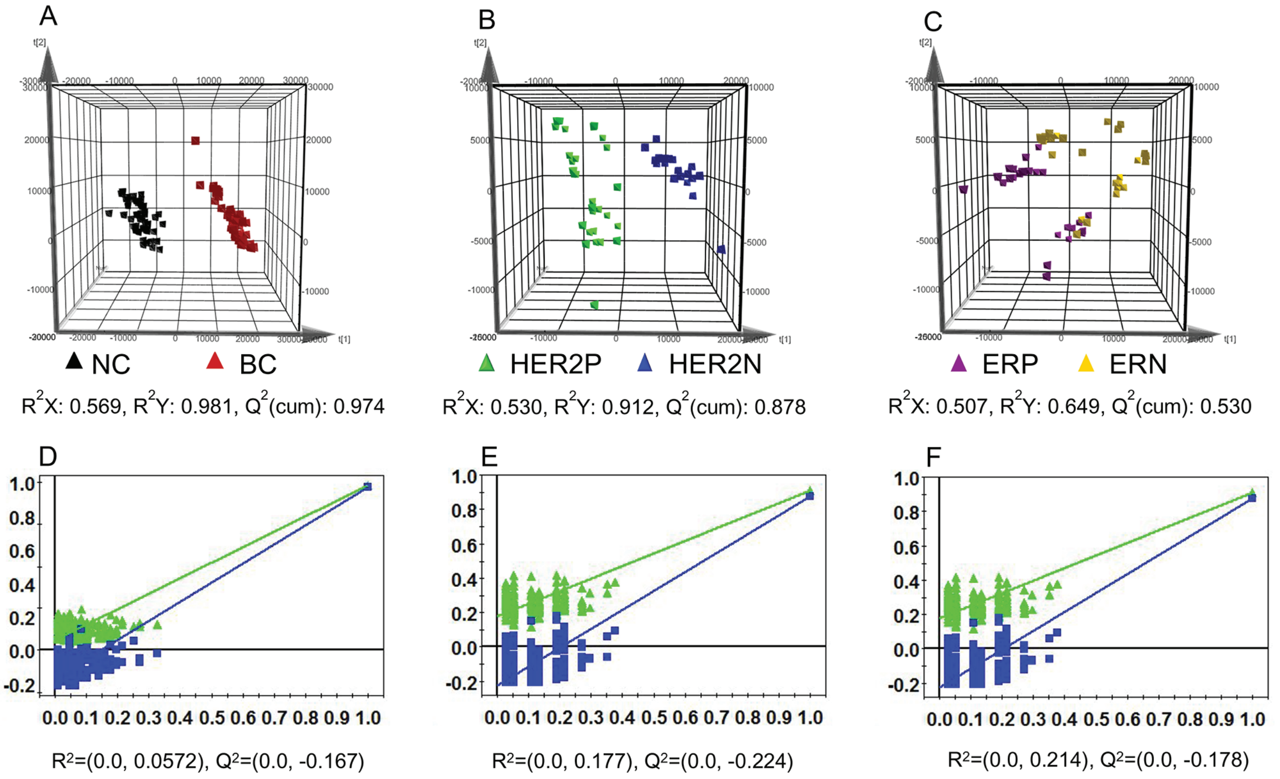

Figure S4: PLS-DA loading plots and chance permutation test obtained from GC-MS. A. Normal control (NC) vs breast cancer (BC) group; **B.** HER2-positive (HER2P) vs HER2-negative (HER2N) BC group; **C.** ER-positive (ERP) vs ER-negative (ERN) group. Black triangle corresponds to NC group, red triangle corresponds to BC group, green triangle corresponds to HER2-positive BC patients, blue triangle corresponds to HER2-negative BC subjects, purple triangle corresponds to ER-positive participants, and yellow triangle corresponds to ER-negative patients. Chance permutation at 200 times for the discrimination between **D.** NC vs BC, **E.** HER2P vs HER2N, **F.** ERP vs ERN.

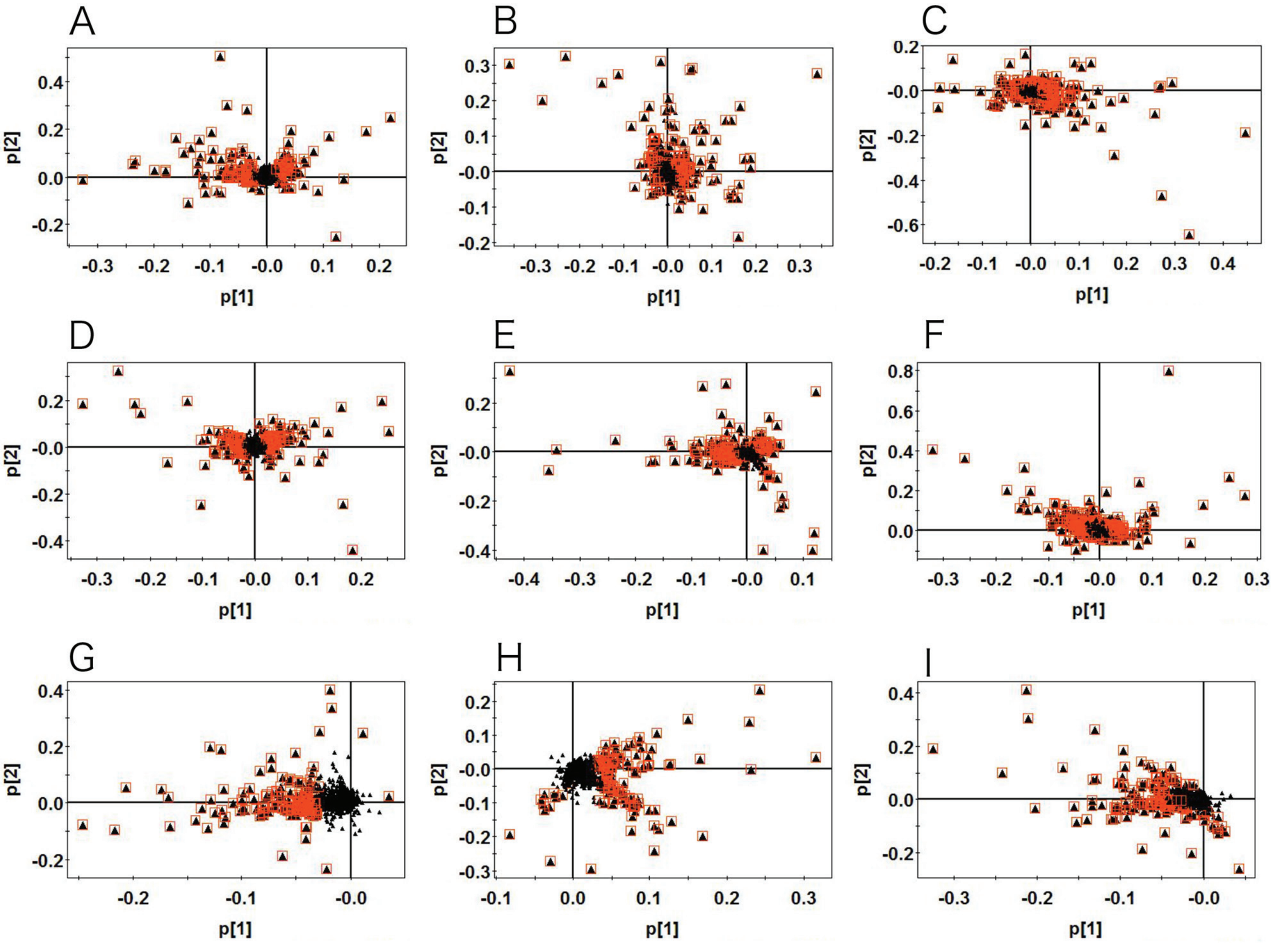

Figure S5: PLS-DA loading plots for the discrimination between normal control (NC) *vs* breast cancer (BC) group; HER2-positive (HER2P) *vs* HER2-negative (HER2N) BC group; ER-positive (ERP) *vs* ER-negative (ERN) group by the peaks obtained from A-C. LC-MS in positive mode, **D-F.** LC-MS in negative mode, and **G-I.** GC- MS. VIP higher than 1 in loading plot was highlighted by red open circles.

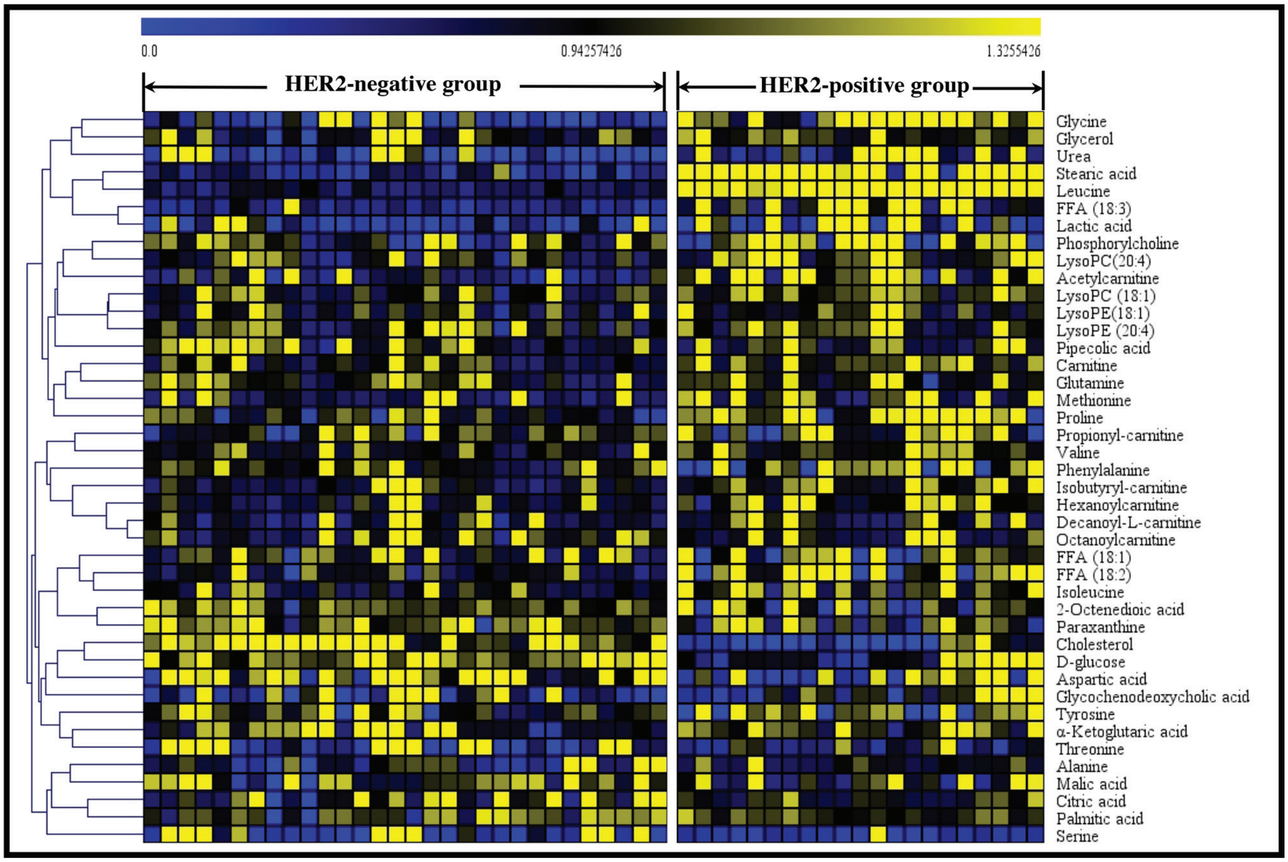

Figure S6: Heatmap of 40 differential metabolites between HER2-positive breast cancer (BC) and HER2-negative BC subjects. The colors from blue to yellow indicate the upregulated level of metabolites.

Table S1: Clinic pathologic characteristics of HER2 positive breast cancer patients enrolled in this study

HER2-positive group (training set)					HER2-positive group (test set)				
Patient	Age	ER status	TNM	Stage	Patient	Age	ER status	TNM	Stage
1	46	N	T2N1M0	IIIB	10	71	P	T2N1M0	IIIB
2	52	N	T2N0M0	IIA	11	52	P	T1N0M0	I
3	43	P	T2N0M0	IIA	15	46	P	T2N0M0	IIA
4	50	N	T2N0M0	IIA	16	45	P	T1N1M0	IIA
5	55	P	T1N0M0	I	20	31	P	T2N1M0	IIIB
6	82	N	T1N1M0	IIA	23	70	P	T2N1M0	IIIB
7	59	N	T2N0M0	IIA	25	47	N	T2N0M0	IIIB
8	73	N	T2N0M0	IIA	26	39	N	T3N1M0	IIIA
9	50	P	T2N0M0	IIA	27	61	N	T1N0M0	I
12	53	N	T2N0M0	IIA	28	65	P	T2N1M0	IIIB
13	45	N	T2N1M0	IIIB	29	56	N	T2N0M0	IIA
14	43	N	T2N0M0	IIA	30	44	P	T1N0M0	I
17	70	N	T1N1M0	IIA	31	60	N	T1N1M0	IIA
18	52	N	T2N0M0	IIA	32	35	P	T2N1M0	IIIB
19	50	N	T3N0M0	IIIB	33	55	N	T1N1M0	I
21	46	N	T2N0M0	IIA					
22	33	N	T2N0M0	IIA					
24	60	N	T2N1M0	IIIB					
34	52	P	T2N0M0	IIA					
35	70	P	T1N1M0	I					
36	56	P	T2N0M0	IIA					

P: positive; N: negative.

Table S2: Clinic pathologic characteristics of HER2 negative breast cancer patients enrolled in this study

HER2-negative group (training set)					HER2-negative group (test set)				
Patient	Age	ER status	TNM	Stage	Patient	Age	ER status	TNM	Stage
1	70	N	T1N0M0	I	31	52	N	T2N0M0	IIA
2	47	N	T2N0M0	IIA	32	67	N	T2N0M0	IIA
3	44	N	T2N0M0	IIA	33	63	N	T1N0M0	I
4	59	N	T2N0M0	IIA	34	46	N	T1N1M0	IIA
5	47	N	T2N1M0	IIIB	35	46	N	T2N0M0	IIA
6	53	N	T2N1M0	IIIB	36	38	N	T2N0M0	IIA
7	58	N	T1N1M0	IIA	37	41	N	T2N0M0	IIA
8	67	N	T2N0M0	IIA	38	56	N	T3N0M0	IIIB
9	40	N	T1N0M0	I	39	43	P	T2N1M0	IIIB
10	35	N	T2N1M0	IIIB	40	63	P	T1N0M0	I
11	55	P	T2N0M0	IIA	41	33	P	T2N1M0	IIIB
12	45	P	T3N0M0	IIIB	42	35	P	T1N1M0	I
13	42	P	T1N1M0	I	43	70	P	T2N0M0	IIA
14	75	P	T1N0M0	I	44	70	P	T1N0M0	I
15	52	P	T1N0M0	I	45	49	P	T1N0M0	I
16	39	P	T1N1M0	IIA	46	35	P	T2N1M0	IIIB
17	62	P	T1N0M0	I	47	44	P	T2N1M0	IIIB
18	62	P	T1N1M0	I	48	43	P	T2N1M0	IIIB
19	43	P	T2N1M0	IIIB	49	37	P	T3N1M0	IIIA
20	60	P	T1N0M0	I	50	59	P	T1N0M0	I
21	45	P	T1N0M0	I	51	77	P	T1N0M0	I
22	73	P	T1N0M0	I	52	45	P	T2N0M0	IIA
23	63	P	T1N0M0	I	53	54	P	T1N0M0	I
24	45	P	T1N0M0	I	54	57	P	T1N0M0	I
25	62	P	T1N0M0	I	55	60	P	T1N0M0	I
26	56	P	T1N0M0	I	56	47	P	T2N0M0	IIA
27	46	P	T2N1M0	IIIB	57	55	P	T2N0M0	IIA
28	62	P	T2N1M0	IIIB	58	62	P	T2N0M0	IIA
29	42	P	T1N0M0	I	59	78	P	T2N0M0	IIA
30	51	P	T2N1M0	IIIB	60	34	P	T2N0M0	IIA

P: positive; N: negative.

Table S3: Differential metabolites identified between breast cancer and normal control plasma and their pathway involved

No.	t_R(min)	*m/z*	Metabolites	Formula	Fold change	*p* value	VIP[a]	Pathway involved
ESI+								
1	0.676	147.0604	Glutamine*	$C_5H_{10}N_2O_3$	1.400	<0.001	0.992	Alanine, aspartate and glutamate metabolism
2	0.733	162.1124	Carnitine	$C_7H_{15}NO_3$	1.428	<0.001	3.935	Fatty acid transportation
3	0.733	116.0711	Proline*	$C_5H_9NO_2$	1.452	0.008	2.378	Arginine and proline metabolism
4	0.959	72.0820	Pyrrolidine	C_4H_9N	0.533	0.004	2.644	Others
5	0.959	118.0869	Valine*	$C_5H_{11}NO_2$	0.733	0.016	4.817	Valine, leucine and isoleucine metabolism
6	1.015	204.1231	Acetylcarnitine	$C_9H_{17}NO_4$	1.338	0.008	3.138	Fatty acid transportation
7	1.015	130.0861	Pipecolic acid	$C_6H_{11}NO_2$	0.621	<0.001	2.650	Protein synthesis, amino acid biosynthesis
8	1.242	150.0548	Methionine*	$C_5H_{11}NO_2S$	0.463	<0.001	2.594	Cysteine and methionine metabolism
9	1.468	182.0815	Tyrosine*	$C_9H_{11}NO_3$	0.498	<0.001	3.787	Aminoacyl-tRNA biosynthesis
10	1.638	218.1378	Propionyl-carnitine	$C_{10}H_{19}NO_4$	0.630	0.040	1.792	Fatty acid transportation
11	1.751	166.0871	Phenylalanine*	$C_9H_{11}NO_2$	0.630	0.022	3.657	Aminoacyl-tRNA biosynthesis
12	1.977	232.1545	Isobutyryl-carnitine	$C_{11}H_{21}NO_4$	0.434	0.003	1.266	Fatty acid transportation
13	2.712	260.1855	Hexanoylcarnitine	$C_{13}H_{25}NO_4$	0.357	<0.001	1.241	Fatty acid transportation
14	3.448	288.2172	Octanoylcarnitine	$C_{15}H_{29}NO_4$	0.224	<0.001	3.182	Fatty acid transportation
15	4.24	502.2933	LysoPE (20:4)	$C_{25}H_{44}NO_7P$	0.903	0.002	1.162	Lysophospholipid catabolism
16	4.296	468.3086	LysoPC(14:0)	$C_{22}H_{46}NO_7P$	0.223	<0.001	3.754	Glycerophospholipid catabolism
17	4.409	316.2485	Decanoyl-L-carnitine	$C_{17}H_{33}NO_4$	0.205	<0.001	3.527	Fatty acid transportation
18	4.466	494.3242	LysoPC(16:1)*	$C_{24}H_{48}NO_7P$	0.395	<0.001	3.696	Glycerophospholipid catabolism
19	4.579	568.3386	LysoPC (22:6)	$C_{30}H_{50}NO_7P$	0.682	0.045	2.576	Glycerophospholipid catabolism

(*continued*)

No.	t_R(min)	*m/z*	Metabolites	Formula	Fold change	*p* value	VIP[a]	Pathway involved
20	4.636	544.3397	LysoPC(20:4)*	$C_{28}H_{50}NO_7P$	0.457	<0.001	7.975	Glycerophospholipid catabolism
21	4.692	454.2957	LysoPE (16:0)	$C_{21}H_{44}NO_7P$	0.468	<0.001	1.512	Lysophospholipid catabolism
22	4.749	184.0734	Phosphorylcholine	$C_5H_{14}NO_4P$	0.521	<0.001	1.303	Glycerophospholipid catabolism
23	4.805	542.3224	LysoPC (20:5)	$C_{28}H_{48}NO_7P$	0.354	0.002	1.497	Glycerophospholipid catabolism
24	4.805	482.3233	LysoPC(15:0)	$C_{23}H_{48}NO_7P$	0.442	<0.001	2.792	Glycerophospholipid catabolism
25	4.862	520.3393	LysoPC (18:2)	$C_{26}H_{50}NO_7P$	0.859	0.002	5.887	Glycerophospholipid catabolism
26	4.862	480.3079	LysoPE(18:1)	$C_{23}H_{46}NO_7P$	0.681	0.008	1.578	Lysophospholipid catabolism
27	4.862	570.3547	LysoPC(22:5)	$C_{30}H_{52}NO_7P$	0.637	0.004	1.226	Glycerophospholipid catabolism
28	5.088	508.3396	LysoPE(20:1)	$C_{25}H_{50}NO_7P$	0.303	<0.001	1.929	Lysophospholipid catabolism
29	5.145	546.3559	LysoPC(20:3)	$C_{28}H_{52}NO_7P$	0.489	<0.001	2.573	Glycerophospholipid catabolism
30	5.654	522.3550	LysoPC (18:1)	$C_{26}H_{52}NO_7P$	0.667	<0.001	8.281	Glycerophospholipid catabolism
31	6.05	548.3704	LysoPC(20:2)	$C_{28}H_{54}NO_7P$	0.385	<0.001	1.636	Glycerophospholipid catabolism
32	6.389	510.3560	LysoPE(20:0)	$C_{25}H_{52}NO_7P$	0.352	<0.001	2.190	Lysophospholipid metabolism
33	6.728	280.2635	Linoleamide	$C_{18}H_{33}NO$	1.154	0.033	3.320	Others
34	7.803	550.3858	LysoPC(20:1)	$C_{28}H_{56}NO_7P$	0.293	<0.001	1.173	Glycerophospholipid catabolism
35	8.029	256.2642	Palmitic amide	$C_{16}H_{33}NO$	0.686	0.001	3.759	Others
**ESI$^-$**								
36	0.688	179.0579	Paraxanthine*	$C_7H_8N_4O_2$	1.214	<0.001	1.002	Purine metabolism
37	0.907	145.0139	α-Ketoglutaric acid*	$C_5H_6O_5$	0.428	<0.001	0.238	Tricarboxylic acid cycle
38	0.914	175.0248	Ascorbic acid*	$C_6H_8O_6$	0.253	<0.001	1.849	Ascorbic acid
39	1.423	133.0139	Malic acid*	$C_4H_6O_5$	0.410	0.037	0.712	Tricarboxylic acid cycle
40	1.536	130.0871	Isoleucine*	$C_6H_{13}NO_2$	1.082	0.031	1.424	Valine, leucine and isoleucine metabolism
41	2.611	171.0659	2-Octenedioic acid	$C_8H_{12}O_4$	1.358	<0.001	3.623	Others

(*continued*)

No.	t_R(min)	*m/z*	Metabolites	Formula	Fold change	*p* value	VIP[a]	Pathway involved
42	3.403	338.9859	Fructose 1,6-bisphosphate	$C_6H_{14}O_{12}P_2$	0.603	<0.001	0.513	Glycolysis metabolism
43	3.573	448.3075	Glycocheno-deoxycholic acid*	$C_{26}H_{43}NO_5$	0.679	0.016	1.834	Bile acid biosynthesis
44	5.092	297.2435	3-Oxooctadecanoic acid	$C_{18}H_{34}O_3$	1.219	<0.001	0.812	Fatty acid metabolism
45	6.684	277.2174	FFA (18:3) *	$C_{18}H_{30}O_2$	5.758	<0.001	0.620	Biosynthesis of unsaturated fatty acids
46	7.532	279.2322	FFA (18:2)*	$C_{18}H_{32}O_2$	1.861	<0.001	1.785	Biosynthesis of unsaturated fatty acids
47	8.833	281.2486	FFA (18:1) *	$C_{18}H_{34}O_2$	1.420	<0.001	0.701	Biosynthesis of unsaturated fatty acids
**GC-MS**								
48	6.299		Lactic acid*	$C_3H_6O_3$	2.401	<0.001	1.057	Glycolysis metabolism
49	6.904		Alanine*	$C_3H_7NO_2$	0.427	<0.001	1.003	Alanine and aspartate metabolism
50	7.117		Glycine*	$C_2H_5NO_2$	0.697	0.003	2.421	Glycine, serine and threonine metabolism
51	7.493		Oxalic acid*	$C_2H_2O_4$	0.262	0.006	2.422	Others
52	8.879		Urea	CH_4N_2O	0.341	<0.001	1.000	Urea cycle
53	9.304		Leucine*	$C_6H_{13}NO_2$	0.847	0.007	1.337	Valine, leucine and isoleucine metabolism
54	9.403		Glycerol*	$C_3H_8O_3$	1.212	<0.001	1.562	Glycerophospholipid metabolism
55	10.612		Serine*	$C_3H_7NO_3$	0.144	0.012	0.678	Glycine, serine and threonine metabolism
56	10.957		Threonine*	$C_4H_9NO_3$	0.084	<0.001	0.702	Glycine, serine and threonine metabolism
57	11.871		Aspartic acid*	$C_4H_7NO_4$	0.332	<0.001	0.967	Alanine, aspartate and glutamate metabolism
58	16.230		Citric acid*	$C_6H_8O_7$	0.790	<0.001	1.418	Tricarboxylic acid cycle
59	16.969		D-fructose*	$C_6H_{12}O_5$	0.273	<0.001	1.393	Others
60	17.034		D-glucose*	$C_6H_{12}O_6$	0.307	<0.001	1.426	Glycolysis metabolism

(*continued*)

No.	t_R(min)	*m/z*	Metabolites	Formula	Fold change	*p* value	VIP[a]	Pathway involved
61	18.045		Palmitic acid*	$C_{16}H_{32}O_2$	1.328	<0.001	1.638	Fatty acid biosynthesis
62	19.862		Stearic acid*	$C_{18}H_{36}O_2$	1.058	<0.001	1.924	Fatty acid biosynthesis
63	22.730		Glycerol 1-palmitate	$C_{19}H_{38}O_4$	1.632	<0.001	1.545	Glycerophospholipid catabolism
64	27.843		Cholesterol*	$C_{27}H_{46}O$	0.719	<0.001	3.006	Hormone biosynthesis and bile acid biosynthesis

* confirmed with reference standards;
[a] fold change >1 indicates that the average normalized peak area ratio in HER2-positive group is larger than that in HER2-negative group;
[b] variable importance in the projection.
